# Using a Performance Based Assessment Tool of Everyday IADL to Determine Driving Risk in Older Adults

**DOI:** 10.1093/geroni/igaa057.2596

**Published:** 2020-12-16

**Authors:** Anne Dickerson

**Affiliations:** East Carolina University, Greenville, North Carolina, United States

## Abstract

For older adults living in rural/suburban communities, driving is often their only means of transportation. Although considered safe drivers, drivers older than 70 years have higher crash rates with fatality rates amplified due to the increased frailty/fragility. However, research evidence clearly indicates that cognitive factors contribute to driving impairment in older adults. Occupational therapists, as experts in observation of functional performance, use the Assessment of Motor and Process Skills (AMPS), for measurement of performance in everyday activities using two scales (motor and process). Previously demonstrated as a sensitive tool for cognitive changes, this presentation will summarize the research outcomes between older adults with cognitive impairment (N=57+) and without (N=53) who completed a comprehensive driving evaluation. Analysis of the two samples using receiving operating curves suggests the AMPS has potentially excellent specificity and sensitivity, specifically AUC = 0.826(0.73-0.92) for motor, AUC = 0.909(0.84-0.98) for process, and AUC = 0.936(0.88-0.99) together. Part of a symposium sponsored by Transportation and Aging Interest Group.

